# Inflammatory Burden and Immunomodulative Therapeutics of Cardiovascular Diseases

**DOI:** 10.3390/ijms23020804

**Published:** 2022-01-12

**Authors:** Ting-Wei Kao, Chin-Chou Huang

**Affiliations:** 1 Department of Internal Medicine, National Taiwan University Hospital, Taipei 100225, Taiwan; twkao315@gmail.com; 2 Division of Cardiology, Department of Medicine, Taipei Veterans General Hospital, Taipei 112201, Taiwan; 3 School of Medicine, National Yang Ming Chiao Tung University, Taipei 112304, Taiwan; 4 Institute of Pharmacology, School of Medicine, National Yang Ming Chiao Tung University, Taipei 112304, Taiwan; 5 Cardiovascular Research Center, National Yang Ming Chiao Tung University, Taipei 112304, Taiwan

**Keywords:** cardiovascular disease, gut microbiota, nephropathy, inflammation

## Abstract

Phenotyping cardiovascular illness and recognising heterogeneities within are pivotal in the contemporary era. Besides traditional risk factors, accumulated evidence suggested that a high inflammatory burden has emerged as a key characteristic modulating both the pathogenesis and progression of cardiovascular diseases, inclusive of atherosclerosis and myocardial infarction. To mechanistically elucidate the correlation, signalling pathways downstream to Toll-like receptors, nucleotide oligomerisation domain-like receptors, interleukins, tumour necrosis factor, and corresponding cytokines were raised as central mechanisms exerting the effect of inflammation. Other remarkable adjuvant factors include oxidative stress and secondary ferroptosis. These molecular discoveries have propelled pharmaceutical advancements. Statin was suggested to confer cardiovascular benefits not only by lowering cholesterol levels but also by attenuating inflammation. Colchicine was repurposed as an immunomodulator co-administered with coronary intervention. Novel interleukin-1β and −6 antagonists exhibited promising cardiac benefits in the recent trials as well. Moreover, manipulation of gut microbiota and associated metabolites was addressed to antagonise inflammation-related cardiovascular pathophysiology. The gut-cardio-renal axis was therein established to explain the mutual interrelationship. As for future perspectives, artificial intelligence in conjunction with machine learning could better elucidate the sequencing of the microbiome and data mining. Comprehensively understanding the interplay between the gut microbiome and its cardiovascular impact will help identify future therapeutic targets, affording holistic care for patients with cardiovascular diseases.

## 1. Background

Inflammation remains a long-standing clinical challenge. An elevated inflammatory status has recently been proposed as an independent risk factor for developing end-organ comorbidities and unfavourable prognosis of various chronic illnesses [1]. Cardiovascular diseases were among those entities proposed to be closely intertwined with inflammation. The heavy clinical burden due to high incidence, leading cause of morbidity, and suboptimal, albeit significantly improved, management of cardiovascular diseases have prompted the development of novel modalities to treat this population. Management against residual inflammatory burden after an index event has gradually been recognised as pivotal to prevent systemic sequelae and improve holistic care. Early meta-analysis in the last decade demonstrated that elevated C-reactive protein (CRP) levels were positively correlated with the risk of coronary artery disease (CAD), ischaemic stroke, and mortality in individuals without underlying vascular diseases [2]. Inflammation was further addressed, giving rise to CAD through its correlation with plaque rupture and thromboembolism [3]. As for terminal consequences, decompensatory remodeling secondary to inflammatory heart leads to heart failure with reduced ejection fraction [4]. Accordingly, multiple clinical trials examined potential strategies to ameliorate inflammation; however, their efficacies remain debatable. Besides anti-inflammatory medications, manipulation of the gut microbiota and its metabolites emerged as novel therapeutic measures. Although promising as the therapeutic target, the mechanism underlying how maintenance of microbiological homeostasis orchestrates cardiovascular physiology warranted further elucidations. Therefore, the objective of this review was to revisit the role of inflammation from a molecular perspective and assess its implications on clinical outcomes in cardiovascular disease.

## 2. Inflammation and Cardiovascular Diseases

### 2.1. Atherosclerosis

Inflammation has been reported to predispose individuals to atherosclerosis [5]. Activated endothelial cells promote the expression of inflammatory markers. Lymphocytes and monocytes migrate toward the endothelium and further infiltrate the arterial wall to induce atherogenesis [6]. The healing of vascular injury depends on the activation of the inflammatory signal, which is the substrate for the formation of atherosclerotic plaques. Historically, this vulnerable plaque theory was conceptualised to link inflammation to vascular events. Toll-like receptor (TLR) 4, nuclear factor κ-light-chain-enhancer of activated B cells (NFκB), and Janus kinase/signal transducer and activator of transcription are inflammation-related pathways that mediate atherosclerosis [7]. The inflammation further facilitates erosion and potential rupturing of the local plaque, resulting in thromboembolic events. From a preventive medicine perspective, amelioration of the inflammatory status has been used to reduce the risk of adverse cardiovascular outcomes and atherothrombosis.

### 2.2. Myocardial Infarction

**Figure 1:**
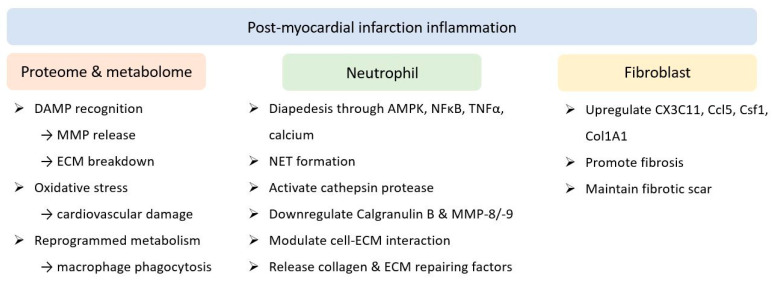
Molecular alterations secondary to inflammation after myocardial infarction. Landscape of proteome and metabolome, as well as neutrophil and fibroblast responses to the inflammatory status. AMPK: AMP-activated protein kinase; DAMP: damage-associated molecular pattern; ECM: extracellular matrix; MMP: matrix metalloproteinase; NET: neutrophil extracellular traps; NFκB: nuclear factor κ-light-chain-enhancer of activated B cells; TNFα: tumour necrosis factor α.

Inflammation after myocardial infarction (MI) is another issue of concern. A previous transcriptome study longitudinally mapped the polarisation of local macrophages [8]. On the first day post MI, initiation of interleukin (IL)-1 signalling, as well as the recognition of damage-associated molecular patterns, was shown to lead to the breakdown of extracellular matrix (ECM) by released matrix metalloproteinase (MMP). Elevated oxidative stress has been proposed to cause cardiovascular damage. In conjunction, reprogrammed metabolism subsequently promoted phagocytosis by resident macrophages. Eventually, transduction of integrin, transforming growth factor β (TGFβ) receptor 1, IL-4 receptor α chain signalling, and signal transducer and activator of transcription 3 signalling resulted in the release of collagen ECM repair factors after 1 week. Neutrophils also participate in the post-MI inflammatory network [9]. AMP-activated protein kinase, NF-κB, tumour necrosis factor (TNF)-α, and calcium signalling were identified as the first responders to diapedesis, followed by the activation of cathepsin protease for apoptosis [10]. These also resulted in left ventricular (LV) wall thickening and hypertrophy. ECM reorganisation and scar formation are known consequences of the inflammation cascade [11]. Additionally, the proteome of neutrophils was altered in response to MI. Expression of calgranulin B and MMP-8/-9 decreased over the time course post MI, while the levels of fibrinogen, fibronectin, vitronectin, MMP-2, TIMP metallopeptidase inhibitor 2, and thrombospondin-2 increased gradually during the same period [12]. Galectin-3, which predominantly modulates cell-ECM interactions, acts as a late responder and increases at the subacute stage for remodelling [13]. By employing peptidylarginine deiminase 4, the formation of neutrophil extracellular traps through chromatin filaments, in conjunction with granular and cytoplasmic components and extracellular vesicles, has been proposed to worsen cardiac inflammation and injury [14]. Fibroblasts were the third player to orchestrate the inflammatory status heralded by MI. Previous studies have reported that *Cx3c11*, *Ccl5*, *Csf1*, and *Tnfrsf9* were among the first genes initially upregulated post MI but regressed during the late course; in contrast, collagen 1A1 increased on post-MI day one and plateaued [15]. In terms of the dynamic role of cardiac fibroblasts, the physiology altered from initial pro-inflammation and pro-angiogenesis to promote fibrosis and ECM composition, and finally, to maintain the fibrotic scar over the infarcted region, thereby reaching new homeostasis [16] (Figure 1).

Specifically, IL-4 is recognised as a brake for post-MI inflammation. Daseke et al. proposed that exogenous IL-4 could antagonise the expression of inflammatory genes, such as *Ccl3*, *Il12a*, *Tnfa*, and *Tgfb1*, in neutrophils and facilitate the expression of the anti-inflammatory profile, Arg1, and Ym1, in macrophages [17]. Phagocytosis by polymorphonuclear leukocytes was found to be induced post MI. These findings further confirmed the role of cardiac macrophages in homeostasis, regeneration, and repair after ischaemic events [18]. The second postulation was established to modulate MMP and its proteolytic products to regulate cardiac remodelling. Iyer et al. demonstrated the protective role of MMP-12 in neutrophil physiology [19]. Based on a juvenile C57BL/6J male mouse model with the left coronary artery ligated to recapitulate acute MI (AMI), inhibition of MMP-12 aggravated LV dilatation and dysfunction. Moreover, pro-inflammatory cytokines, IL1r1, IL6ra, IL11, and Cxcr5 were upregulated, whereas CD44 level, caspase 3 cleavage, and hyaluronic acid degradation were attenuated up to 7 days post MI. MMP-9 is secreted by the AMP-activated protein kinase, protein kinase C, c-Jun N-terminal kinase, and p38 mitogen-activated protein kinase/extracellular signal-regulated kinase pathways. These signalling transductions from macrophages and neutrophils are initiated by inflammatory mediators following MI. Activated MMP-9 proteolyses ECM components, which promote the polarisation of resident inflammatory cells for phagocytosis and apoptosis. Additionally, LV remodelling, along with geographical adaptation and scar formation, are subsequent consequences [20].

## 3. Molecular Mechanism Underlying Inflammation

The molecular mechanism governing the role of inflammation in cardiovascular diseases has garnered considerable research attention. The inflammatory status can be activated by diverse aetiologies, such as myocardial ischaemia, viral myocarditis, hypertrophic or autoimmune cardiomyopathy, and genetic diseases. The injured myocardium triggers both innate and adaptive immunity [21]. In addition, cholesterol crystals, neutrophil extracellular traps, atheroprone flow, and hypoxia contribute to the activation of the inflammatory status. Binding of pathogen-associated molecular patterns or damage-associated molecular patterns to pattern recognition receptors, including TLRs and nucleotide oligomerisation domain (NOD)-like receptors, on local cardiomyocytes or residing immune cells, induces downstream signal transduction pathways. Eventually, the cascade leads to the upregulation of chemokines and inflammatory cytokines. The most commonly involved players are the IL family and TNF. Transcriptional profiling has also reported that the genetic landscape associated with inflammation is altered in patients with heart failure and myocardial ischaemia [22]. In addition, macrophages and neutrophils are recruited collectively to initiate adaptive immunity.

In recent decades, identifying biochemical parameters to assess the inflammatory status has also been revolutionised. CRP is the most accepted biomarker and is widely used in current practice. The upstream molecules were subsequently found to be clinically relevant. These molecules directly respond to external stimuli, transduce signalling intracellularly, and orchestrate liver events for eventual CRP production. IL-1β, IL-6, IL-18, and TNF are among the pro-inflammatory cytokines, while IL-1 receptor antagonists, IL-10, IL-19, and IL-33 antagonise inflammation [23]. These markers further foster the corresponding pharmaceutical agents to manage the inflammatory status. Classic examples include methotrexate that specifically targets IL-6, a newly developed monoclonal antibody canakinumab, and anakinra, in conjunction with colchicine, pinpointed IL-1β, and tocilizumab, which regulates intermediate signal transduction [24].

Ferroptosis induced by excessive oxidative stress is another central player amid inflammation. As one of the non-apoptotic regulated necrosis and unlike other immunologically silent programmed cell death, ferroptosis was recently proposed to induce inflammation [25]. Excessive ferroptosis was established to aggravate cardiovascular morbidities, whereas attenuating ferroptosis by antioxidants was proposed to improve prognosis [26]. Nemade et al. manipulated cardiomyocytes differentiated from human induced pluripotent stem cells, which were then treated with etoposide. Antagonising p53-mediated ferroptosis, in addition to apoptosis, suppressed cardiotoxicity [27]. To investigate the underlying molecular mechanism, a rodent model with aortic binding versus sham operation demonstrated that the TLR-4/NADPH oxidase 4 pathway is involved in autophagy and ferroptosis during cardiac failure [28]. Cardiac injury secondary to sepsis was proposed to be a consequence of ferroptosis through the heme oxygenase-1 pathway [29]. In addition, ferroptosis has been addressed as a potential therapeutic target, as it predominantly occurs during the reperfusion stage rather than during acute infarction. Inhibition of ferroptosis was found to ameliorate reperfusion injury after MI by reducing endoplasmic reticulum stress and the ATF4-CHOP pathway [30]. Targeting the TLR-4, Trif, and type 1 interferon pathways also reduced cardiomyocyte injury at the reperfusion stage after heart transplantation [31]. Still, the exact role of ferroptosis with regards to inflammation and its correlation with immunogenicity mandated future investigations.

Determining the phenotype of patients with atherosclerosis after pharmaceutical or angiographic intervention is the cornerstone of personalised medicine. Conventionally, the cholesterol burden is well-known to be intertwined with cardiovascular morbidities. However, in clinical trials involving lipid-lowering agents, the residual inflammatory risk, defined as a serum CRP level > 2 mg/L, emerged to be equally important. According to early reports, for example, CARE [32], A to Z [33], and REVERSAL [34] optimised clinical outcomes were observed in patients who met the target therapeutic goal of both cholesterol and CRP levels. In conjunction with hypercholesterolaemia, these studies underscored the association between elevated inflammatory status and subsequent atherosclerosis, as well as thromboembolic sequelae. In the JUPITER trial with 17,802 otherwise healthy subjects with elevated high-sensitivity CRP > 2 mg/L, the administration of rosuvastatin dramatically decreased major adverse cardiovascular events (MACEs) [35]. This was the earliest evidence to indicate inflammation as a cardiovascular risk factor. Nevertheless, some researchers have argued that reduced cholesterol levels afforded the remarkable benefit, despite being within the normal range at baseline, rather than the modest anti-inflammatory effect. Indeed, as demonstrated by Pravastatin or Atorvastatin Evaluation and Infection Therapy-Thrombolysis in Myocardial Infarction 22 (PROVE IT-TIMI 22) study, 29% of the 3745 individuals with acute coronary syndrome exhibited a high inflammatory status, albeit under statin treatment [36]. In comparison, only 14% of this cohort failed to reach the goal of an optimised cholesterol level ˂ 70 mg/dL. A similar condition was observed in the IMProved Reduction of Outcomes: Vytorin Efficacy International Trial (IMPROVE-IT) randomised trial. The residual risks of inflammation, cholesterol, or both were 33%, 14%, and 14%, respectively [37]. As for the real-world database regarding patients with chronic atherosclerosis, the United States Variation in Recovery: Role of Gender on Outcomes of Young AMI Patients (VIRGO) registry documented a surprisingly high proportion (60%) of the statin-treated population presenting a residual inflammatory burden, with females exhibiting a higher predisposition [38].

## 4. Pharmaceutical Advancements

### 4.1. Colchicine

To achieve adequate anti-inflammation, the indication for colchicine was revisited. As a traditional and orally available medication against the NOD-like receptor protein 3 (NLRP3) inflammasome, the use of colchicine has been broadened, despite notorious gastrointestinal adverse effects. Further trials with colchicine were undertaken to evaluate cardiovascular impacts in various clinical settings. In the Colchicine Cardiovascular Outcomes trials (COLCOT) [39] study with 4745 participants sustaining recent MI within one month, the introduction of low-dose colchicine was associated with a significant reduction in subsequent ischaemic cardiovascular events. Based on this finding, in the Low-Dose Colchicine 2 (LoDoCo2) trial [40], 5522 patients with chronic CAD were included to receive colchicine (0.5 mg) per day or placebo. At the 28.6-month follow-up, the composite of MACE was remarkably attenuated following the administration of anti-inflammatory agents. Additionally, multiple attempts have been made to delineate the cardiovascular benefit by ameliorating periprocedural vascular injury and inflammation secondary to percutaneous coronary intervention (PCI). The inflammatory risk in patients undergoing PCI is clinically relevant. A large retrospective study assessing 7026 subjects after intervention proposed that persistently high CRP levels were significantly correlated with all-cause mortality and MI risk [41]. Deftereos et al. examined 151 patients with ST-elevation MI (STEMI) as a pilot study. Colchicine was loaded after PCI and was maintained for 5 days [42]. Although the peak levels of cardiac enzymes and infarction size by cardiac magnetic resonance imaging were reduced, this anti-inflammatory medication failed to decrease the occurrence of MACEs. Conversely, post-procedural administration of colchicine showed adequate safety and tolerance, as well as successfully reduced the rate of re-hospitalisation in the Low-Dose Colchicine after Myocardial Infarction (LoDoCo-MI) trial with 237 enrolled patients [43]. The timing of drug administration was subsequently adjusted. In the COLIN trial [44], Akodad et al. evaluated colchicine within 24 h of STEMI onset and maintained it for the subsequent 30 days. However, the incidence of MACEs showed no significant changes. As in the COLCHICINE-PCI randomised trial [45], administration of 1.8 mg colchicine before the procedure successfully decreased IL-6 and CRP levels. Nevertheless, at one month, the risk of PCI-related myocardial injury, mortality rate, nonfatal MI, and target vessel revascularisation remained unaltered. This addressed whether cardiac physiology would actually respond to anti-inflammatory regimens. However, interest in colchicine-mediated immunomodulation has persisted. Drug combinations have surfaced as the mainstream in contemporary trial design. In the Colchicine and Spironolactone in Patients with MI/SYNERGY Stent Registry (CLEAR SYNERGY) trial, anti-inflammatory agents in conjunction with diuretic use were intended to improve post-MI prognosis. In the SYNERGY trial, an estimated 7000 patients with STEMI who were referred for PCI were enrolled [46]. This study pioneered the attempt to include immunomodulatory agents in goal-directed medical therapy.

### 4.2. Interleukin-1 Antagonist

Meanwhile, other anti-inflammatory agents, apart from colchicine, were assessed for their cardiovascular effects. In the Canakinumab Anti-inflammatory Thrombosis Outcome Study (CANTOS) trail [47], 10,061 post-MI subjects with elevated CRP were enrolled. Targeting IL-1β with canakinumab markedly reduced the rate of recurrent cardiovascular events. Methotrexate, a widely used anti-inflammatory agent in rheumatological diseases, was evaluated but failed to improve the clinical efficacy of secondary prevention in patients with stable atherosclerosis. In the Cardiovascular Inflammation Reduction Trial (CIRT) [48], 4786 individuals with previous MI or multivessel coronary disease, concomitant diabetes mellitus (DM), or metabolic syndrome were enrolled. At the 2.3-year follow-up, methotrexate did not reduce the composite rate of cardiovascular mortality, nonfatal MI, or nonfatal stroke. Anakinra-mediated IL-1 blockade has been evaluated for its clinical efficacy as a potential therapeutic target. Abbate et al. conducted a pilot study with 30 patients sustaining STEMI [49]. The first dose of anakinra 100 mg was administered within 24 h of primary PCI and continued for 14 days. A significant decrease in serum CRP level and the rate of mortality or new-onset heart failure was documented, with no reduction in the level of CK-MB and MACE incidence. In the MRC-ILA Heart Study [50], a total of 182 patients with acute non-ST-elevation MI (NSTEMI) were enrolled for performing a randomised trial. The first dose was administered promptly in the first 2 days of symptom onset and lasted for the subsequent 14 days. Levels of IL-6 and CRP were successfully reduced following anakinra administration but unexpectedly rebounded after drug discontinuation. Nevertheless, the rate of MACE revealed no significant change at 30 days and was increased in the following year, primarily driven by the increased incidence of MI. Meanwhile, targeting cellular immunity by antagonising anti-CD20 has been attempted. Rituximab in patients with acute ST-elevation MI (RITA-MI) is an ongoing phase 1/2a trial, enrolling 24 individuals with CAD after effective coronary intervention [51]. The safety profile and B-cell depleting effect of post-PCI single-dose rituximab were well demonstrated, whereas the alteration of inflammatory marker expression and its impact on clinical outcomes need to be elucidated.

### 4.3. Interleukin-6 Antagonist

In addition, IL-6 has emerged as a novel therapeutic target. Mendelian randomisation analysis reported that the genetic variant in the IL-6 receptor was associated with a lifelong risk of CAD [52]. Seven single nucleotide polymorphisms in the proximal or within the IL-6 receptor transcription domain were identified by genome-wide sequencing in 204,402 Europeans [53]. Downregulation of the IL-6 pathway by genetic modification was associated with significantly lower levels of CRP and correlated with a reduced risk of cardiovascular disease and prolonged longevity [54]. Based on the low-density lipoprotein (LDL)-receptor-deficient murine model, an anti-mouse IL-6 receptor antibody was administered to mice fed a high-fat diet for one week. Surprisingly, a pronounced improvement in atherosclerotic lesions was observed [55]. As for clinical translation, stratified subgroup analysis of the CANTOS study with 4848 subjects exhibiting stable CAD suggested that post-treatment IL-6 levels were positively correlated with the risk of cardiovascular sequelae [56]. To antagonise IL-6 activity, a single dose of tocilizumab 280 mg was administered to 117 subjects with NSTEMI prior to coronary intervention [57]. In conjunction with CRP, troponin levels were decreased but failed to curtail the incidence of MACE. Attempts to develop and employ novel agents to antagonise IL-6 signalling are underway. A trial evaluating the reduction in inflammation in patients with advanced chronic renal disease using antibody-mediated IL-6 inhibition (RESCUE) is an ongoing phase II trial intended to demonstrate the cardiovascular benefit of IL-6 inhibition by ziltivekimab [58]. In total, 264 individuals with moderate to severe chronic kidney disease and elevated CRP levels were enrolled. The preliminary outcome revealed a reduction in inflammatory and thrombotic markers in a dose-dependent manner without affecting the cholesterol level. Remarkably, CRP showed a two-fold reduction following ziltivekimab administration when compared with that following canakinumab in the CANTOS trial. To further demonstrate the efficacy of ziltivekimab and extend its indication, the Ziltivekimab Cardiovascular Outcomes study (ZEUS) is another ongoing trial targeting individuals with atherosclerotic cardiovascular disease, significant renal insufficiency, and high CRP levels. This event-driven study intended to demonstrate that IL-6 inhibition effectively attenuated the occurrence of cardiovascular events [59]. Focus on this specific patient group was suggested to identify eligible medications for managing inflammation. As colchicine is relatively contraindicated in patients with renal insufficiency, who also endorsed a higher cardiovascular risk, ziltivekimab might be the next clinical candidate that mitigates the inflammatory status without altering cholesterol levels (Table 1).

## 5. The Role of Gut Microbiota

A disorganised profile of the intestinal microorganism was proposed to be interrelated with inflammation under the context of cardiovascular diseases [60]. Amelioration of inflammatory status by modulating the gut microbiota composition is expected to serve as a new modality. Translocation of the microorganisms and shedding of the bacterial wall compound can contribute to the inflammatory status. Additionally, not only dysbiosis but also the altered metabolism gives rise to inflammation. First, the short-chain fatty acid generated by anaerobic fermentation of fibre is considered pivotal for initiating inflammatory signalling [61]. Second, butyrate is another end metabolite of fermentation that had been identified to govern regulatory T cells for orchestrating inflammation and recognised prerequisite to maintain the intestinal barrier [62]. A recent study with metagenome sequencing indicated the expression of the butyrate-encoding gene was negatively correlated with the level of inflammatory markers, which was proposed secondary to the altered abundance of *Roseburia* and *Eubacterium* in subjects with carotid atherosclerosis [63]. Third, lipopolysaccharide (LPS) shed from Gram-negative bacteria was also pointed out as the bridge of remodeled microorganisms to inflammation and eventually attributing to the pathogenesis of cardiovascular diseases. The enteric LPS invades systemic circulation and ignites inflammation after being recognised by TLRs. The elevated endotoxin level and pro-inflammatory status eventually compromise cardiac and vascular function [64].

Moreover, trimethylamine N-oxide (TMAO) was identified as another chief transmitter from dysbiosis to the inflammatory status impacting cardiovascular manifestations based on the metabolomic perspective. The quantity of TMAO was determined by age, body mass index, and especially the gut microbiome composition. A Cleveland cohort study with 530 individuals with suspected acute coronary syndrome and 1683 patients from a multi-centre Swiss population suggested that trimethyllysine and/or TMAO were of prognostic significance [65]. The abundance of these markers predicted short- and long-term all-cause mortality rates, as well as cardiovascular events. TMAO also participates in atherosclerosis. Oral intake of L-carnitine from red meat facilitated the release of trimethylamine by the intestinal microbiome, which was transformed to TMAO by flavin-containing monooxygenase in the liver and eventually altered cholesterol metabolism to give rise to atherosclerosis [66]. By employing liquid chromatography and tandem mass spectrometry for quantification, a high level of TMAO was associated with a predisposed risk of MACEs [67].

Clinically, the impacts of inflammatory status induced by gut dysbiosis have been displayed in a wide spectrum of cardiovascular disease. Our previous review summarised the correlation between gut microbiota and inflammation in patients with metabolic syndrome [68]. Luedde et al. observed a significantly lower diversity index in 20 individuals with heart failure secondary to ischaemic or dilated cardiomyopathy, which was marked by the depletion of *Coriobacteriaceae*, *Erysipelotrichaceae*, and *Ruminococcaceae* [69]. Moreover, the outgrowth of *Campylobacter*, *Shigella*, *Salmonella*, *Yersinia enterocolitica*, and *Ruminococcus gnavus* was observed in the faecal sample of patients with chronic heart failure as compared with healthy subjects by metagenomic and metabolomics profiling [70,71]. The composition of the microbiome is also related to electrical conduction. Inspired by an early study indicating that elevated levels of inflammatory cytokines can be detected in patients with atrial fibrillation (AF) [72], investigating the gut microbiome has gained momentum. Literature has addressed that gut metabolites, including lipopolysaccharide and indoxyl sulfate, can increase the instability of myocardial substrate and predispose the onset of AF [73]. Jacob et al. summarised that the residual inflammatory burden was correlated with new-onset AF after cardiac operation [74]. Moreover, a gut-derived metabolite was proposed to predict the risk of MACE in the context of AF [75]. In a prospective study, faecal samples from 34 admitted patients with AF were harvested and compared with those from healthy counterparts. Despite similar diversity, the number of species was significantly lower in the AF arm. Furthermore, a reduced quantity of *Enterobacter* was observed, where the abundance of *Parabacteroides*, *Lachnoclostridium*, *Streptococcus*, and *Alistipes* were remarkably elevated. These pathologic alterations were proportional to the New York Heart Association functional class and correlated with the initiation of inflammatory status [76]. In addition, the activated atrial NLRP3-inflammasome predisposed the onset of age-related AF triggered by accumulated serum polysaccharide [77], which also predicts the occurrence of MACEs in subjects with AF [75]. Zhang et al. further demonstrated that the introduction of young microbiota from 6~8-month-old donors effectively attenuated the development of arrhythmia in aged 16~18-year-old recipients based on this rodent model [78].

To validate whether remodeling of dysbiosis would improve cardiovascular outcomes, Chen et al. developed a Western diet-fed and LDL receptor double knockout mouse model [79]. Using an in vitro screening protocol, cyclic D, L-α-peptides were found to alter the composition of the gut microbiome to that observed on administering a low-fat diet. As for a plant-based diet, anti-inflammatory and cardioprotective effects with abounded Bifidobacterium and Lactobacillus by polyphenol was another classic example demonstrating altered microbiological signature by diet was of clinical significance [80,81]. Furthermore, the transcriptome landscape was reprogrammed to downregulate the expression of pro-inflammatory cytokines. Antagonism of IL-1β, IL-6, and TNF-α work synergistically with modified levels of short-chain fatty acids and bile acids. Both the serum level of total cholesterol and the development of atherosclerotic plaque were lowered. This was the first proof-of-concept experiment to demonstrate the feasibility of suppressing atherosclerosis by modulating the gut microbiome. Recently, the application of bioinformatics with artificial intelligence to interpret the inflammatory landscape has opened a new chapter. Using the Gene Expression Omnibus database, Chen et al. identified 57 differentially expressed genes predominantly intertwined with the crosstalk between cytokines, chemokines, and TNF signalling [82]. Guo et al. used bioinformatics and data mining to investigate the alteration of gene expression in AMI and unstable angina. Interestingly, 20 genes related to inflammation have been identified for the development of cardiac ischaemic events [83]. In addition, an integrated network analysis was established to propel advancements in genetic enquiries and to discover novel molecular therapies. Several long non-coding RNAs were proposed by nucleotide isolation and sequencing to play a central role in the diagnosis of MI, myocardial fibrosis, and heart failure [84]. The encoding of IL-8 and IL18R1 was addressed to be related to atherosclerosis, as well as serve as a potential diagnostic marker. Other genes, including *IL1R2*, *IRAK3*, *LRG1*, and *PLAC4*, have diagnostic implications [85]. In addition to three-dimensional printing and in silico manufacturing, machine learning was integrated to mine the microbiome database, optimise analytical yield, and facilitate the design of microbiome-directed and/or molecular-driven therapeutics [86].

## 6. Gut-Cardio-Renal Triplet

**Figure 2:**
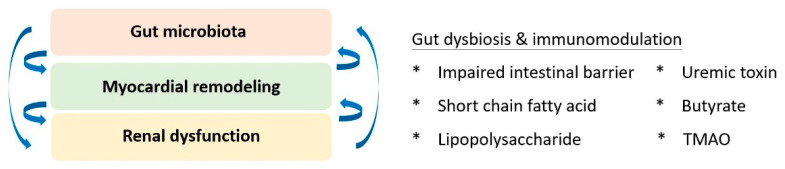
The gut-cardio-renal triplet. The gut microbiome and the pathophysiology of cardiac and renal systems are mutually interactive and dependent on the inflammatory response. TMAO: trimethylamine-N-oxide.

Based on the accumulating evidence of mutual interplay, the triplet of the gut-cardio-renal axis was conceptualised to modify traditional cardiorenal syndrome (Figure 2). The homeostasis of gut microbiota and its immunoregulatory effects are pivotal for maintaining the physiology of both the cardiac and renal systems. Regarding kidney physiology, impaired intestinal barrier function, microbial dysbiosis, compromised immunity, and toxin production were primary factors that correlated with altered gut microbiota and renal dysfunction [87]. Short-chain fatty acids have been identified as key players in immune regulation, signalling of G-protein-coupled receptors, and antagonists of histone deacetylases for epigenetic modulation [88]. Additionally, the pathogenesis of cardiorenal dysfunction implicates the effect of uraemic toxins, including indoxyl sulfate, p-cresyl sulfate, p-cresol, phenylacetic acid, indole-3-acetic acid, homocysteine, hippuric acid, and phenol [89]. Collectively, the search for an upstream therapeutic target for better-integrated care remains a priority. Linaclotide, a guanylate cyclase C agonist, was identified as a potential candidate. Low-dose linaclotide was suggested to prevent and manage cardiorenal syndrome by downregulating plasma TMAO, uraemic toxin, and colonic claudin-1 levels [90].

## 7. Conclusions

In conclusion, the residual inflammatory burden indicates a novel phenotype in patients with cardiovascular disease. In addition to traditional cardiovascular risk factors, the status of inflammation has been established to develop cardiovascular dysfunction, as well as compromise prognosis. With advances in our understanding of molecular networks, the generation of next-generation immunomodulatory agents specifically targeting new therapeutic targets identified via molecular investigations remains the current priority. Mechanism-based rationale to comprehend inflammation in the context of concomitant cardiovascular disease is currently the mainstream in bench studies. A better depiction of the interplay between inflammatory status and target organs constructed the gut-cardio-renal triplet. On the other end of the spectrum, future clinical trials with extended ethical backgrounds will facilitate the generalisation of these medications to orchestrate the inflammatory status from a clinical bedside perspective. Artificial intelligence with machine learning will be a novel modality for data mining and to assess the metabolome, transcriptome, and proteome. Specific management of signalling molecules will pave the way for individualised prescription and precision medicine.

## Figures and Tables

**Table 1:** Pharmaceutical management of inflammatory status and corresponding cardiovascular effects.

Trial/Author, Year	Cohort	Dosage, Timing	Outcomes	Ref
Colchicine				
COLCOT, 2019	4745 subjects with recent MI	0.5 mg QD	Colchicine reduced further ischaemic events at 22.6 months.	[39]
LoDoCo2, 2020	5522 subjects with chronic CAD	0.5 mg QD	Colchicine remarkably attenuated MACEs at 28.6 months.	[40]
Deftereos, 2015	151 subjects with STEMI	2 mg loading after PCI, then 0.5 mg BID for 5 days	Colchicine decreased accumulative level of CK-MB and infarction size after primary PCI against STEMI.	[42]
LoDoCo-MI, 2019	237 patients admitted for acute MI	0.5 mg QD	Colchicine reduced neither the decrease nor the absolute level of CRP at 30 days.	[43]
COLIN, 2017	44 patients underwent PCI against STEMI	1 mg QD following acute MI, lasting 1 month	Colchicine failed to reduce the peak level of CRP.	[44]
COLCHICINE-PCI, 2020	714 patients referred for possible PCI	1.8 mg acute pre-procedural	Colchicine reduced the serum level of IL-6 and CRP but failed to decrease PCI-related MI.	[45]
CLEAR SYNERGY, ongoing	Patients referred for PCI against STEMI	0.5 mg BID	To validate the efficacy of colchicine against MACEs.	[46]
Interleukin-1 antagonist				
CANTOS, 2017	10,061 patients with prior MI and CRP ≧ 2 mg/L	Canakinumab (50, 150, 300 mg) per 3 months subcutaneously	Canakinumab effectively reduced the recurrent cardiovascular events.	[47]
CIRT, 2019	4786 patients with prior CAD and DM or MetS	Methotrexate 15–20 mg per week	Methotrexate did not improve composite cardiovascular outcomes.	[48]
Abbate, 2013	30 patients with STEMI	Anakinra 100 mg loading acutely after PCI, then maintained for 14 days	Anakinra reduced CRP level, mortality rate, and new-onset heart failure.	[49]
MRC-ILA Heart Study, 2015	182 patients with NSTEMI	Anakinra 100 mg within 2 days of symptom onset, lasting 14 days	Anakinra reduced CRP and IL-6 levels, both of which rebounded after drug discontinuation.	[50]
Interleukin-6 antagonist				
Kleveland, 2016	117 patients with NSTEMI	Tocilizumab 280 mg, single dose	Tocilizumab curtailed inflammation and PCI-related troponin rise.	[57]
RESCUE, 2021	264 patients with CKD and elevated CRP	Ziltivekimab 7.5, 15, or 30 mg per 4 weeks, up to 24 weeks	Ziltivekimab attenuated the expression of inflammatory markers and thromboembolism.	[58]
ZEUS, ongoing	Patients with atherosclerosis, renal insufficiency, and elevated CRP	Ziltivekimab 15 mg for up to 4 years	Aimed to demonstrate that ziltivekimab would reduce cardiovascular events.	[59]

BID: twice per day; CAD: coronary artery disease; CK-MB: creatine kinase-MB; CKD: chronic kidney disease; CRP: C-reactive protein; IL: interleukin; DM: diabetes mellitus; MACE: major adverse cardiovascular events; MetS: metabolic syndrome; MI: myocardial infarction; PCI: percutaneous coronary intervention; QD: once per day; STEMI: ST-segment elevation myocardial infarction.

## Data Availability

The data set supporting the content of this article has been published elsewhere.
